# Antemortem detection of *Mycobacterium bovis* in nasal swabs from African rhinoceros

**DOI:** 10.1038/s41598-023-50236-8

**Published:** 2024-01-03

**Authors:** Rebecca Dwyer, Carmel Witte, Peter Buss, Robin Warren, Michele Miller, Wynand Goosen

**Affiliations:** 1 Division of Molecular Biology and Human Genetics, Faculty of Medicine and Health Sciences, Department of Science and Innovation – National Research Foundation Centre of Excellence for Biomedical Tuberculosis Research, South African Medical Research Council Centre for Tuberculosis Research, Stellenbosch University, P.O. Box 241, Cape Town, 8000 South Africa; 2 The Center for Wildlife Studies, P.O. Box 56, South Freeport, ME 04078 USA; 3 Veterinary Wildlife Services, Kruger National Park, Private Bag X402, Skukuza, 1350 South Africa

**Keywords:** Tuberculosis, Infectious-disease diagnostics

## Abstract

*Mycobacterium bovis* (*M. bovis*) infection has been identified in black (*Diceros bicornis*) and white (*Ceratotherium simum*) rhinoceros populations in Kruger National Park, South Africa. However, it is unknown whether *M. bovis* infected rhinoceros, like humans and cattle, can shed mycobacteria in respiratory secretions. Limited studies have suggested that rhinoceros with subclinical *M. bovis* infection may present minimal risk for transmission. However, recent advances that have improved detection of *Mycobacterium tuberculosis* complex (MTBC) members in paucibacillary samples warranted further investigation of rhinoceros secretions. In this pilot study, nasal swab samples from 75 rhinoceros with defined infection status based on *M. bovis* antigen-specific interferon gamma release assay (IGRA) results were analysed by GeneXpert MTB/RIF Ultra, BACTEC MGIT and TiKa–MGIT culture. Following culture, speciation was done using targeted PCRs followed by Sanger sequencing for mycobacterial species identification, and a region of difference (RD) 4 PCR. Using these techniques*,* MTBC was detected in secretions from 14/64 IGRA positive rhinoceros, with viable *M. bovis* having been isolated in 11 cases, but not in any IGRA negative rhinoceros (n = 11). This finding suggests the possibility that MTBC/*M. bovis*-infected rhinoceros may be a source of infection for other susceptible animals sharing the environment.

## Introduction

Mammalian tuberculosis (TB) is a chronic progressive disease that is primarily caused by infection with *Mycobacterium bovis* (*M. bovis*). Between 2016 and 2019, *M. bovis* infection was discovered in black (*Diceros bicornis*) and white (*Ceratotherium simum*) rhinoceros in Kruger National Park (KNP), South Africa^1–3^. These findings were followed by a population-wide study in KNP (of 437 rhinoceros sampled between 2016–2020), which revealed that 15.4% (95% CI; 10.4–21.0%) of rhinoceros were infected with *M. bovis,* based on a combination of mycobacterial culture and antigen-specific interferon gamma release assay (IGRA) results^4^. The relatively high prevalence suggests that rhinoceros in KNP are regularly infected with *M. bovis*. In the cases in which infection was diagnosed postmortem, lesions and *M. bovis* isolation were primarily associated with the respiratory tract^2,5–8^. However, it is unknown whether infected rhinoceros, like humans and cattle, can shed mycobacteria in respiratory secretions^9,10^.

Although mycobacterial culture is the gold standard for diagnosing *Mycobacterium bovis* infection, it is widely recognized that antemortem detection can be insensitive, due to intermittent shedding, inability to obtain appropriate samples, and harsh decontamination processes, resulting in false negative diagnosis of infected individuals^11,12^. Therefore, diagnostic tests based on mycobacterial antigen-specific immunological responses, such as the delayed hypersensitivity response in the tuberculin skin test or in vitro cytokine release assays, are commonly used to identify *M. bovis* infected hosts^11,13^. Currently, the only available antemortem test that has been validated for detection of *M. bovis* infection in rhinoceros is the QuantiFERON TB Gold Plus Mabtech equine interferon gamma release assay (IGRA)^1,14^. Although tests based on host responses are useful for screening and surveillance, direct detection of *M. bovis* in secretions, such as respiratory samples, is crucial for understanding the epidemiology of TB and determining risk of spread from infected animals^15–18^.

Some studies have suggested that subclinically infected rhinoceros are unlikely to shed *M. bovis*, and, therefore, may present minimal risk for transmission^6,19^. Conventional mycobacterial culture methods, applied to bronchoalveolar lavage samples, recovered *M. bovis* in only 1 out of 60 samples collected over 20 months from three experimentally-infected white rhinoceros^6^. It is unknown whether the low recovery rate was due to the true absence of *M. bovis* in lavage samples, or low sensitivity of culture methods^6^. However, recent advances have improved detection of MTBC in paucibacillary samples and warrant further investigation of rhinoceros secretions^4,8,20^.

Applications of novel enhanced mycobacterial culture techniques and improved PCR-based speciation have led to increased *M. bovis* detection in wildlife^13,16,18,21–24^. Use of cationic D-enantiomer peptide supplementation and modified decontamination methods (TiKa-MGIT) have facilitated *Mycobacterium tuberculosis* complex (MTBC) isolation from paucibacillary tissue and respiratory samples^21,22^. Culture-independent direct detection, using Cepheid’s GeneXpert MTB/RIF Ultra qPCR assay (Ultra), supports same day MTBC DNA detection from a variety of animal specimens^16,18,24–27^. Development of PCRs priming highly conserved genomic region flanking areas within the *rpoB* and *hsp65* genes with high genomic diversity provides new methods for detection and speciation of *Mycobacteria* spp^23,24,28,29^. The value of applying these techniques has already been demonstrated in studies of wildlife TB^24^. Therefore, these may also be valuable for detecting *M. bovis*, especially in antemortem samples, from suspected infected rhinoceros, providing key information for future epidemiological studies. In this study, the overall aims were 1) to determine whether *M. bovis* (DNA and viable bacilli) could be detected using novel direct detection approaches for nasal swabs from suspected *M. bovis* infected (IGRA positive) rhinoceros in KNP, and 2) to calculate the proportion of IGRA positive rhinoceros with *M. bovis* present in nasal secretions to elucidate the potential for mycobacterial shedding.

## Materials and methods

### Study population

Black (n = 39) and white (n = 472) rhinoceros in KNP were opportunistically sampled during immobilisations performed as part of management and veterinary activities between January 2020 and April 2022. Demographic characteristics were documented during immobilisation; these included species (black or white rhinoceros), sex (male or female) and age class, which was estimated by veterinary staff during capture and summarised as follows: calf (0–2 years); subadult (> 2 to 7 years); adult (> 7 years).

**Figure 1:**
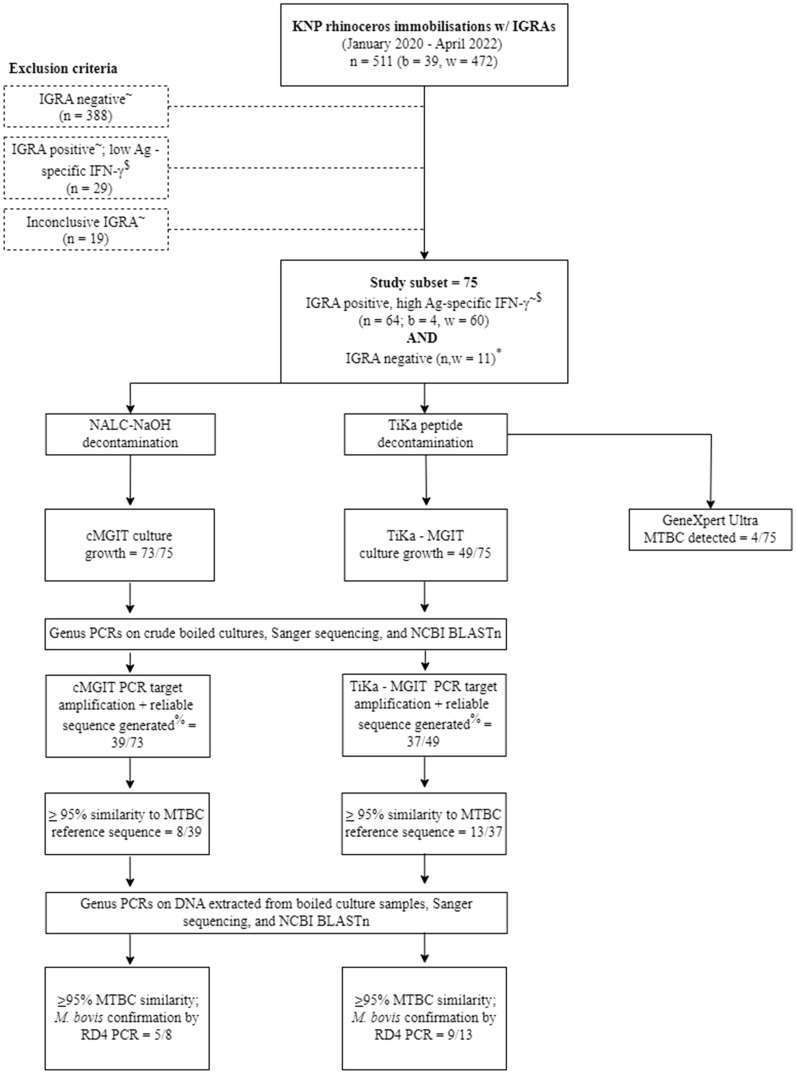
Flow chart for identifying the study population and methods pipeline for detection of MTBC in nasal swabs from African rhinoceros. KNP, Kruger National Park; b, black rhinoceros; w, white rhinoceros; IGRA, interferon-gamma release assay; Ag, antigen; IFNg, interferon-gamma; cMGIT, conventional BACTEC MGIT Mycobacterial Growth Indicator Tube culture method; TiKa–MGIT, TiKa decontamination and growth supplement enhanced MGIT culture; NCBI BLASTn, National Centre for Biotechnology Information Basic Local Alignment Search Tool (nucleotide); MTBC, *Mycobacterium tuberculosis* complex; *M. bovis*, *Mycobacterium bovis*; *RD4*, (genetic) Region of Difference 4. ^~^A rhinoceros was classified as IGRA positive or negative, according to previously defined cutoff values^1,4^. It was considered IGRA negative if it had a TB Ag-specific IFNg response ≤ 21 pg/mL, a mitogen IFNg response ≥ 21 pg/mL, and a nil IFNg response ≤ 21 pg/mL. It was classified as IGRA positive if it had a TB Ag-specific IFNg response > 21 pg/ml. Individuals who could not be defined as IGRA positive or negative according to the described case definitions were considered inconclusive and excluded. ^$^IGRA positive rhinoceros were included in the study subset for examination of nasal swabs based on the magnitude of their Ag-specific IFNg responses. Of the 93 IGRA positive individuals, the 64 with the highest Ag-specific IFNg responses (ultimately, all with Ag-specific [IFNg] ≥ 40 pg/ml) were included in the study subset. The remaining 29 IGRA positive individuals with lower Ag-specific IFNg (ultimately, all with Ag-specific [IFNg] < 40 pg/ml) were excluded from the study subset. *A small selection of IGRA negative rhinoceros were included for comparison.

Routinely collected samples included heparinized whole blood and nasal swabs, which were processed as described below, and in Fig. 1. A subset of nasal swab samples was chosen based on rhinoceros interferon gamma release assay (IGRA) results. The IGRA was applied for measurement of antigen-specific interferon gamma (IFNg) release in whole blood that had been stimulated using the QuantiFERON Gold Plus (QFT) platform (Qiagen, Venlo, Limburg, Netherlands), along with an anti-equine interferon gamma ELISA (Mabtech Ab, Nacka Strand, Sweden)^1,4^. Specifically, antigen-specific IFNg concentrations in this study were determined by subtracting the concentration in the nil tube from the TB2 antigen tube, as recommended by the manufacturer. This study subset consisted of 75 total rhinoceros: 64 that were IGRA positive (suspect *M. bovis* infected) and 11 that were IGRA negative (suspect uninfected). The number of IGRA negative animals included in the study subset was determined by available resources for testing and randomly selected from all IGRA negative rhinoceros. A detailed explanation of the selection criteria for this subset is provided in Fig. 1.

### Nasal swab sample collection and processing

A single nasal swab (FLOQswab, Copan Diagnostics, Murrieta, California, USA) was collected from each rhinoceros at the time of blood collection. The swab was immediately transferred into 1 ml of sterile saline and immediately frozen, then transported to Stellenbosch University. Nasal swabs were thawed and further processed for direct detection of MTBC, using the Ultra qPCR assay and two different mycobacterial culture methods followed by molecular identification of mycobacterial DNA, as shown in Fig. 1.

#### Conventional and modified mycobacterial cultures

Frozen nasal swab samples were thawed and 2 ml sterile phosphate (PO_4_) buffer was added and mixed in the BSL-3 laboratory. Each sample was split into three equal aliquots (Fig. 1). The first aliquot (1 ml supernatant) was stored at − 80 °C and was not used further for this study. The second aliquot (nasal swab tip and 1 ml supernatant) was decontaminated with N-acetyl L-cysteine sodium hydroxide (NALC-NaOH) and processed for culture using the BACTEC Mycobacteria Growth Indicator Tube (MGIT) 960 TB System (Becton Dickinson, Franklin Lakes, New Jersey, USA), as previously described^12,22,23^. The third 1 ml aliquot was processed for culture using a modified version of the conventional MGIT (cMGIT) system, TiKa-MGIT (TiKa Diagnostics, London, United Kingdom)^22^. Briefly, samples were transferred to 30 ml sterile tubes containing 10 ml TiKa-Kic decontamination agent (TiKa Diagnostics) and incubated overnight (for a minimum of 20 h) at 37ºC. Thereafter, samples were centrifuged at 3000 × g for 20 min and the supernatant was discarded. The cell pellets were resuspended in 1.6 ml PO_4_ buffer and thoroughly mixed before 1 ml of each sample was removed and kept aside for testing (post-TiKa decontamination) in the GeneXpert MTB/RIF Ultra qPCR assay (Cepheid, Sunnyvale, California, USA). The remaining 600 µl was used to inoculate MGIT tubes containing 800 µl BD BACTEC MGIT 960 Supplement Kit (Becton Dickinson) and 8.5 µl TiKa growth supplement B (TiKa Diagnostics). All culture samples were incubated in the BACTEC MGIT 960 TB System incubator at 37˚C for a minimum of 56 days. Samples with no growth after 56 days were regarded as culture negative, and no further downstream analysis was performed. One ml aliquots of culture growth positive samples were removed from the bottom of the MGIT tube, where bacterial growth had settled as seen by visible turbidity, boiled for 30 min at 99 °C and then removed from the BSL-3 facility for downstream testing.

#### Molecular detection of MTBC DNA using PCR and amplicon sequencing

Specialised PCRs, priming highly conserved genomic regions within the *hsp65* and *rpoB* genes^28,29^, were performed to screen boiled culture aliquots to determine presence of *Mycobacterium spp*., as previously described^23,24^. Presence of amplified products with the correct target sizes (*hsp65*: ± 439 bp and *rpoB*: ± 764 bp) was confirmed using 1% agarose gel electrophoresis, followed by gel imaging using the ChemiDoc M.D. Universal Hood III Gel Documentation System (Bio-Rad Laboratories, Hercules, California, USA). Amplicons were sent to the Central Analytical Facility (CAF), Stellenbosch University, for Sanger sequencing. Sequence pairwise alignments were performed using A plasmid Editor (ApE; Version 3.1.3)^30^. Generated consensus sequences were analysed using the National Centre for Biotechnology Information (NCBI) nucleotide Basic Local Alignment Search Tool (BLASTn)^31^ to find sequence alignment matches in the NCBI database^32^.

Culture isolates, that produced consensus target sequences with ≥ 95% shared MTBC reference sequence identity, were selected for further investigation. Additional 1 ml aliquots of these cultures were boiled at 99˚C for 30 min and removed from the BSL-3 facility. Total DNA was extracted from each sample aliquot using the QIAGEN DNeasy Blood and Tissue Kit (Qiagen, Hilden, Germany), as previously described. The *hsp65* PCR was repeated, using extracted DNA instead of boiled culture as the template. Presence of the *hsp65* amplicon was confirmed using 1% agarose gel electrophoresis, and amplicons were sent to CAF for Sanger sequencing. Sequence pairwise alignments and analysis using the NCBI database were completed as described above. Presence of MTBC in the extracted DNA samples was determined according to a threshold of 95% similarity between the query (sample DNA) amplicon sequence, and the MTBC reference target sequence. Extracted DNA samples identified to have MTBC present underwent an additional PCR targeting the genetic region of difference 4 (*RD4*) to confirm the presence of *M. bovis,* as previously described^33^. A 25 µl reaction contained 12.5 µl Q5 High-Fidelity 2X Master Mix (New England Biolabs), 0.5 µl of each 50 µM primer stock solution, 6 µl sterile, nuclease free water, and 5 µl extracted DNA. PCR cycling conditions were as follows: 1 cycle initial denaturation at 98˚C for 15 min, followed by 40 cycles of denaturation (98˚C for 30 s), annealing (62˚C for 1 min) and elongation (72˚C for 1 min). Final elongation took place at 72˚C for 2 min. Presence of the amplified products was confirmed by 1% agarose gel electrophoresis, followed by gel imaging using the ChemiDoc M.D. Universal Hood III Gel Documentation.

#### GeneXpert MTB/RIF ultra qPCR assay

The Ultra qPCR assay (Cepheid) was used for direct detection of MTBC DNA in the remaining 1 ml sample aliquots kept aside after overnight TiKa decontamination (Fig. 1). One ml of GeneXpert sample reagent was added to the 1 ml sample aliquot. The mixture was incubated for 15 min at room temperature, followed by vortexing for 10 s, and incubated for another 5 min before a final vortex for 10 s. The total volume (2 ml) of lysed sample was transferred to the Ultra cartridge sample chamber, loaded into the GeneXpert instrument and PCR performed, according to manufacturer’s guidelines^18,25^. Possible result outputs included MTB not detected, MTB detected high/medium/low/very low/trace. Detection of any MTB (trace amounts or greater) was interpreted as an Ultra positive result.

### Data analyses

The proportion of IGRA positive individuals was determined for the entire study population. Demographic characteristics of the rhinoceros subset selected for further investigation were summarized. Frequency distributions of MTBC detected in nasal swabs from IGRA positive and negative groups were determined across each of the three different detection methods (Ultra qPCR, cMGIT culture/PCR, TiKa-MGIT culture/PCR). In addition, parallel interpretation was used to estimate the proportion of IGRA positive, as well as IGRA negative, rhinoceros with MTBC detected in nasal swabs using any of the three methods; 95% confidence intervals were calculated using the Agresti-Coull method^34^.Test agreement of these three methods within the IGRA positive group was evaluated using a Cohen’s kappa statistic, and qualitatively interpreted^35^. All statistical calculations were performed in R (version 4.3.0, R Core Team).

### Ethics

All procedures including immobilisation of animals and blood and swab collection were undertaken by South African Veterinary Council—registered wildlife veterinarians for management or other veterinary procedures unrelated to this study. Procedures were carried out according to the SANParks Standard Operating Procedures for the Capture, Transportation and Maintenance in Holding Facilities of Wildlife.Ethical approval for this project was granted by the Stellenbosch University Animal Care and Use Committee (ACU-2020-0966, ACU-2020-19019, ACU-2021-19019, ACU-2022-19019) and the Stellenbosch University Biological and Environmental Safety Research Ethics Committee (SU-BEE-202122561; SU-BES-202322561). Section 20 approval was issued by the South African Department of Agriculture, Land Reform and Rural Development (DALRRD; 12/11/1/7/2, 12/11/1/7/2A (JD)).

An approved Biomaterial Transfer Agreement (BMTA 005/22; 011/19) was obtained from South African National Parks (SANParks) which includes evaluation by their Animal Care and Use Committee. A Threatened or Protected Species (TOPS) permit was obtained through the South African Department of Environmental Affairs (DEA Standing Permit S02556; S65805 and DEA Registration Certificate 29416; 02256).

All procedures involving potentially infectious material (e.g. mycobacterial culture) were performed in a Biosafety Level 3 (BSL3) facility that is certified for compliance under the Directorate Animal Health (DAH) of DALRRD. All methods were performed in accordance with the relevant guidelines and regulations as described by Stellenbosch University’s Animal Care and Use Committee and SANParks. ARRIVE guidelines for reporting animal research have been followed (https://arriveguidelines.org/).

## Results

The *M. bovis* infection status, based on IGRA results, was determined for 492 of the 511 (472 white, 39 black) sampled rhinoceros. A total of 93 out of 492 individuals (19%; 95% CI: 16–23%) were IGRA positive, which included 87 white rhinoceros and 6 black rhinoceros. Demographic characteristics of the rhinoceros included in the study subset (64 IGRA positive and 11 IGRA negative) are outlined in Table 1. A total of 14 of the 64 (22%; 95% CI: 13–34%) study rhinoceros that tested IGRA positive had MTBC detected in their nasal swab by at least one of the methods (parallel interpretation), with viable *M. bovis* isolated in 11/64 (17%; 95% CI: 10–28%) cases. Individual test results are shown in Table 2. In summary, 4/64 (6%) swabs were MTBC positive by Ultra, 5/64 (8%) were *M. bovis* positive by cMGIT, and 9/64 (14%) were *M. bovis* positive by TiKa-MGIT. None of the swab samples had MTBC detected using all three methods; however, four samples had MTBC detected by two methods—specifically, three swabs were *M. bovis* positive by cMGIT/PCR and TiKa-MGIT/PCR, and one swab was MTBC positive by Ultra and TiKa-MGIT/PCR (with confirmation of *M. bovis* in the latter). None of the nasal swabs from IGRA negative rhinoceros had MTBC detected by any of the applied methods. Importantly, none of the *rpoB* target amplicon sequences from any of the samples had ≥ 95% similarity to any analogous MTBC sequence in the NCBI BLASTn database; therefore, identification of possible MTBC presence in the samples was determined solely based on a ≥ 95% similarity of the sample *hsp65* target amplicon sequences to the MTBC reference *hsp65* sequence (NCBI sequence ID: CP074075.1: 528,755 to 529,195) in the NCBI BLASTn database. In the IGRA positive group (n = 64), there was fair test agreement between MTBC detection by cMGIT and TiKa-MGIT culture methods (κ = 0.3648, *p* = 0.04), and no test agreement between cMGIT culture and Ultra (κ =  − 0.0746, *p* = 0.003) and Tika culture and Ultra (κ = 0.0737, *p* = 0.6), respectively.

**Table 1:** Summary of demographic characteristics of rhinoceros selected as study population from which nasal swabs were tested for direct detection of MTBC (n = 75).

Demographic characteristics	Number of IGRA positive rhinoceros (% of total in category) n = 64	Number of IGRA negative rhinoceros (% of total in category) n = 11
Species		
White rhinoceros (*C. simum*)	60 (94%)	11 (100%)
Black rhinoceros (*D. bicornis*)	4 (6%)	0 (0%)
Sex		
Male	33 (52%)	4 (36%)
Female	31 (48%)	7 (64%)
Age		
Calf (0 to 2 years)	9 (14%)	3 (27%)
Subadult (> 2 to 7 years)	9 (14%)	3 (27%)
Adult (> 7 years)	46 (72%)	5 (46%)

Antigen-specific interferon gamma (IFN-ɣ) concentrations were measured in QuantiFERON TB Gold Plus stimulated heparinized whole blood plasma using the Mabtech equine IFN-ɣ ELISA, and those with antigen-specific interferon gamma concentrations < 21 pg/ml, mitogen interferon gamma concentration ≥ 21 pg/ml, and nil interferon gamma concentration ≤ 21 pg/ml were considered IGRA negative; IGRA positive rhinoceros included in this cohort had antigen-specific IFN-ɣ concentrations ≥ 40 pg/ml.

**Table 2:** Demographic characteristics and direct detection test results of nasal swabs for 14 interferon gamma assay (IGRA) positive rhinoceros in which MTBC/*M. bovis* was detected by at least one of the applied methods.

Rhinoceros ID	Species	Sex	Age	TB2–Nil [IFN-γ] (pg/ml	Ultra result (+ / −)*	*M. bovis* present in culture sample, determined by *hsp65* PCR followed by *RD4* PCR confirmation using extracted DNA	
						cMGIT	TiKa-MGIT
1	White rhinoceros	Female	Adult	80	−	Yes	Yes
2	White rhinoceros	Male	Adult	128	−	Yes	Yes
3	White rhinoceros	Male	Adult	146	−	Yes	Yes
4	Black rhinoceros	Male	Adult	163	−	Yes	No
5	White rhinoceros	Female	Adult	57	−	Yes	No
6	Black rhinoceros	Male	Calf	40	−	No	Yes
7	White rhinoceros	Female	Adult	95	−	No	Yes
8	White rhinoceros	Female	Subadult	40	−	No	Yes
9	White rhinoceros	Male	Adult	87	+	No	Yes
10	White rhinoceros	Male	Subadult	63	−	No	Yes
11	White rhinoceros	Male	Subadult	470	−	No	Yes
12	White rhinoceros	Male	Adult	66	+	No	No
13	White rhinoceros	Male	Adult	89	+	No	No
14	White rhinoceros	Female	Adult	412	+	No	No
Total MTBC positive by method					4	5	9

*Positive Ultra results in this study were all in trace amounts.

Mycobacteria other than MTBC were present in the nasal swab samples, based on DNA amplification of conserved regions of *hsp65* and *rpoB* in initial PCRs. Although Sanger sequencing and sequence alignment matches in the NCBI database identified NTM species, no further characterization was performed in this study.

## Discussion

*Mycobacterium tuberculosis* complex organisms were detected in nasal swabs using the applied direct detection methods from 14/64 (22%, 95% CI: 13–34%) of the IGRA positive rhinoceros tested, with viable *M. bovis* isolated in 11/64 (17%; 95% CI: 10–28%) cases. Similarly, MTBC has been directly detected in nasal samples from various species with confirmed infection. For example, in a small group (n = 12) of African buffaloes (*Syncerus caffer*) with culture-confirmed *M. bovis* infection, MTBC DNA was detected in 5 of 12 (41%) nasal swabs^16^. In a separate study of a human population with culture-confirmed *M. tuberculosis* infection (n = 80), *M. tuberculosis* DNA was detected in oral swabs from 29 (36.3%) individuals^36^. In a KNP population of African wild dogs (*Lycaon pictus*) with immunological sensitisation to *M. bovis* (determined using an IGRA) (n = 136), *M. bovis* was only recovered by conventional mycobacterial culture in 4 oronasal samples^17^. However, to our knowledge, this is the first report of *M. bovis* detection in nasal (swab) samples from African rhinoceros. These findings demonstrate that MTBC/*M. bovis* can be sporadically detected in nasal cavities of infected African rhinoceros.

The presence of *M. bovis* in nasal secretions of rhinoceros could be indicative of several possible scenarios. Since these respiratory samples were obtained from rhinoceros that were free-ranging in an *M. bovis* endemic area and share the environment with infected hosts^37,38^, the presence of *M. bovis* in these samples may result from environmental contamination or exposure. Alternatively, *M. bovis* may be present in respiratory secretions of a truly infected rhinoceros that is shedding. In this study, MTBC DNA and/or viable *M. bovis* were only detected in nasal secretions from study individuals with immunological evidence of infection (IGRA positive), and not from any IGRA negative individuals, which supports the hypothesis that at least some of the IGRA positive individuals were truly infected and may be shedding mycobacteria. This raises concern that *M. bovis* infected rhinoceros may be a source of exposure for other susceptible animals sharing the environment^15,17,39^. Inclusion of a larger sample size of IGRA negative rhinoceros in future studies could better inform this hypothesis.

This study applied multiple techniques for direct detection of MTBC, including the culture-independent GeneXpert MTB/RIF Ultra qPCR assay, followed by two mycobacterial culture methods, including cMGIT culture and TiKa-MGIT culture with subsequent application of genus PCRs and Sanger sequencing to identify presence of MTBC DNA. The cMGIT culture and TiKa-MGIT cultures only agreed 36% of the time (*p* = 0.04); this reflects the substantially higher recovery of MTBC by TiKa-MGIT culture (n = 9 positive) compared to cMGIT culture (n = 5 positive). This is concordant with findings from other comparative studies thus far, which support the use of TiKa agents to enhance culture recovery from paucibacillary specimens, irrespective of sample type^22,24^.

Both culture methods identified more MTBC positive samples than the Ultra, and there was no agreement between the Ultra and the two culture methods, respectively. In Ultra positive, culture negative samples (Table 2), this could reflect the presence of only non-viable bacilli in some of the samples, which can be detected using Ultra, but not culture. Conversely, *M. bovis* was isolated using culture from several samples that tested Ultra negative (Table 2). This may reflect the ability of culture (and to a greater extent, TiKa-MGIT culture) to select for and enhance viable *M. bovis* to a level that is detectable by molecular methods, even in the presence of other environmental microorganisms (including non-tuberculous mycobacteria (NTMs)) that may otherwise obscure the presence of *M. bovis* and confound culture independent detection using Ultra^22^. Alternative explanations for the culture positive, but Ultra negative results include the presence of PCR inhibitors, or low numbers of bacilli, below the limit of detection for the Ultra, but sufficient to grow in culture. A previous study has shown that the Ultra’s limit of detection for *M. tuberculosis* was 2 colony forming units (CFU) per ml, versus 30 CFU/ml for *M. bovis*^18^.

While both *rpoB* and *hsp65* PCRs were initially applied post-culture in this study, evidence suggested that the *rpoB* target selected was less specific to MTBC. Since the *rpoB* primers targeted highly conserved genomic regions in *Mycobacterium* spp., both NTMs and MTBC DNA would have been amplified in the PCR. However, low levels of MTBC may have been obscured in the Sanger sequencing alignment matches if there was a high abundance of NTMs with *rpoB* target sequences^29^. The *hsp65* target appeared to be more robust and better suited for detection of MTBC, with MTBC only detected in culture isolates based on this marker in combination with Sanger sequencing and a NCBI BLASTn database search, followed by species confirmation of *M. bovis* using the *RD4* PCR. However, since the *RD4* PCR was developed for use with tissues (higher mycobacterial load) compared to paucibacillary respiratory samples^33^, it is recommended that future studies explore the combinational use of different specialized PCRs for MTBC detection, rather than a single target, to increase confidence in results.

A limitation of this study was that rhinoceros were selected based on IGRA results. The presence of false IGRA positive rhinoceros, due to cross-reactive host immune response to NTMs, could not be ruled out. Previous studies in African buffaloes have reported the high diversity of NTMs present in respiratory samples^23^. A study investigating *M. bovis* shedding in African wild dogs in KNP described a low percentage of *M. bovis* positive cultures (3.1–3.5%) of respiratory samples from IGRA positive individuals^17^, like findings in the current study. However, Parsons et al. (2017) described a waning in QFT IGRA responses in three *M. bovis* experimentally infected white rhinoceros over time, with only a low IFNg in vitro response to purified protein derivative of *M. avium*^19^. This suggests that a positive QFT IGRA response in rhinoceros is more likely due to infection with MTBC rather than NTM.

Since only a single nasal swab was obtained from each study individual, there was a limited sample volume available for examination using direct detection methods. Furthermore, examination of a single swab sample per individual provided only a single time point representation of the possible shedding status. Sampling of a rhinoceros cohort at multiple time points may provide a better indication of the potential for mycobacterial shedding by rhinoceros over the course of *M. bovis* infection.

An additional limitation was the small number of IGRA negative (suspect uninfected) individuals included in this study for comparison. These samples were chosen randomly from the larger IGRA negative cohort, and the number was based on available resources for testing the samples. To increase confidence that *M. bovis* detection in rhinoceros nasal samples is indicative of true infection and possible shedding, a larger subset of IGRA negative individuals should be examined.

Finally, nasal swabs are much easier and cheaper to obtain from a large number of rhinoceros than bronchoalveolar/tracheal lavage samples. However, the nasal swab sample type is more likely to have environmental contamination. This could include contamination with environmental MTBC, which may introduce a false positive result that is suggestive of shedding when this is not the case. Alternatively, it may be contamination by NTMs or other environmental microorganisms that can obscure the presence of MTBC in the sample, resulting in misclassification of the sample as MTBC negative. Examination of respiratory lavage samples using the same techniques applied in this study, albeit only feasible in a smaller study population, may increase confidence that any *M. bovis* detected is truly indicative of shedding, and should minimise misclassification of *M. bovis* positive respiratory samples as negative.

## Conclusion

This study showed that viable *M. bovis* may be detected in nasal secretions from rhinoceros with immunological evidence of infection. The KNP rhinoceros population may have an important role in persistence and transmission of *M. bovis* in the KNP system. Larger scale studies of antemortem respiratory samples of KNP rhinoceros, employing direct detection approaches with additional MTBC markers, and sequencing techniques with higher depth of coverage, may be warranted. In addition, comparison of *M. bovis* whole genome sequences isolated from rhinoceros and other reservoir hosts, such as African buffaloes, would provide valuable insight into the epidemiology of TB in the KNP system. Future research would enable better characterisation of possible shedding patterns, presence of other MTBC members, contribution of the KNP rhinoceros population to persistence of *M. bovis* in the ecosystem, and the possible transmission risk associated with the translocation of KNP rhinoceros for management and conservation purposes.

## Data Availability

Summarised data are included in the manuscript. The reference DNA sequence dataset analysed during the current study is available in the NCBI BLASTn database [https://blast.ncbi.nlm.nih.gov/Blast.cgi]. Rhinoceros-specific data are highly sensitive due to the ongoing crisis of poaching for rhinoceros horn and data restrictions apply.
